# Enhancing the Mechanical Properties of AM60B Magnesium Alloys through Graphene Addition: Characterization and Regression Analysis

**DOI:** 10.3390/ma17184673

**Published:** 2024-09-23

**Authors:** Song-Jeng Huang, Jeffry Sanjaya, Yudhistira Adityawardhana, Sathiyalingam Kannaiyan

**Affiliations:** Department of Mechanical Engineering, National Taiwan University of Science and Technology, Taipei 106336, Taiwan; sgjghuang@mail.ntust.edu.tw (S.-J.H.); yudhis1996@gmail.com (Y.A.)

**Keywords:** graphene reinforcement, mechanical properties, regression analysis

## Abstract

The light weight and high strength of magnesium alloys have garnered significant attention, rendering them suitable for various applications across industries. Nevertheless, to meet industrial requirements, the mechanical properties must be improved. This investigation explores the potential of graphene addition to enhance the mechanical properties of AM60B magnesium alloy. Tests were conducted on samples with different weight percentages (wt.%) of graphene (0 wt.%, 0.1 wt.%, and 0.2 wt.%) using stir casting. The elongation and tensile strength of the composite materials were also assessed. The phase composition, particle size, and agglomeration phenomena were analyzed using characterization techniques such as X-ray diffraction, optical microscopy, and SEM-EDS. The yield strength of the magnesium alloy was enhanced by approximately 13.4% with the incorporation of 0.1 wt.% graphene compared to the alloy without graphene. Additionally, an 8.8% increase in elongation was observed. However, the alloy tensile properties were reduced by adding 0.2 wt.% graphene. The tensile fractography results indicated a higher probability of brittle fracture with 0.2 wt.% graphene. Furthermore, regression analysis employing machine learning techniques revealed the potential of predicting the stress–strain curve of composite materials.

## 1. Introduction

Magnesium alloys are known for their light weight and high specific strength and have garnered significant attention for various applications in industries such as automotive, aerospace and biomedical [1,2,3]. Among these alloys, AM60B stands out due to its favorable mechanical properties and corrosion resistance, making it a popular choice for structural applications. However, to meet the evolving demands of these industries, further enhancement of their mechanical properties is necessary [4].

Recent advancements have shown that the incorporation of nano-reinforcements such as graphene can significantly improve the mechanical performance of metal matrix composites (MMCs) [5]. Graphene, which exhibits exceptional mechanical strength and electrical properties, has emerged as a promising reinforcement material [6]. The addition of graphene to magnesium alloys enhances their properties such as yield strength, tensile strength, and ductility [7]. However, the optimal concentration of graphene and its interaction with the matrix material require careful investigation to maximize these benefits without introducing adverse effects like agglomeration [8].

The main hypothesis of this study is that small additions of graphene (specifically 0.1 wt.%) can significantly improve the mechanical properties of AM60B magnesium alloy by enhancing dislocation movement and strengthening grain boundaries. However, we expect that at higher concentrations (such as 0.2 wt.% and above), graphene may lead to agglomeration, which would offset the reinforcing benefits and result in a deterioration of the alloy’s mechanical performance. This hypothesis is supported by conflicting results in the literature: some studies suggest that increased graphene content leads to better material properties [9], while others have expressed that excess graphene may result in decreased ductility due to its tendency to agglomerate [10].

In this study, the mechanical properties of AM60B alloy with varying graphene contents (0 wt.%, 0.1 wt.%, and 0.2 wt.%) were evaluated through yield strength, tensile strength, and elongation measurements. The primary objective was to determine the optimal graphene concentration that maximizes mechanical performance without introducing adverse effects like brittleness. Furthermore, this study aims to provide a detailed understanding of the metallurgical mechanisms underlying the performance differences across these compositions, focusing on how graphene affects dislocation behavior, load transfer mechanisms, and grain boundary interactions.

To achieve these objectives, the study employs a combination of advanced characterization techniques, including X-ray diffraction (XRD) for phase identification, scanning electron microscopy (SEM) for microstructural analysis, and energy-dispersive X-ray spectroscopy (EDS) to examine the elemental distribution of the composites [11,12]. In addition to the experimental analysis, regression modeling was applied to predict the stress–strain behavior of the composites based on the observed microstructural features and mechanical properties [13]. By integrating experimental data with regression analysis, this study not only investigated the optimal graphene content for strengthening AM60B but also contributed to understanding how nano-reinforcements can be effectively utilized in metal matrix composites.

## 2. Materials and Methods

The base material selected for this study was AM60B magnesium alloy, known for its excellent balance of strength, ductility, and corrosion resistance, making it ideal for various structural applications. The material was purchased from Guangyu Technology Co., Ltd. (Shenzhen, China). The chemical composition of AM60B is given in Table 1. Table 2 details the compositions of the alloying elements used, expressed as percentages (wt.%).

To further enhance the mechanical properties of AM60B, graphene was selected as the reinforcement material owing to its superior mechanical properties. The graphene reinforcement was purchased from Jiehan Technology Corporation (Taichung, Taiwan), Taiwan. Composites were prepared with three different graphene contents: 0 wt.% (as a control), 0.1 wt.%, and 0.2 wt.%. This approach was used to investigate the effects of small additions of graphene on the mechanical performance and microstructure of the alloy, allowing for a comprehensive analysis of how graphene influences the material’s overall behavior. 

The specific weight percentages of graphene (0.1 wt.% and 0.2 wt.%) were selected based on prior research, which indicated that higher graphene content (above 1 wt.%) could negatively affect the material’s properties. Even in earlier studies, small amounts of graphene were reported to have potentially adverse effects on composite behavior [14,15]. As a result, lower percentages of graphene were chosen to avoid these issues. Small additions of graphene can significantly enhance mechanical properties, although the effect varies depending on the matrix material. This study specifically investigates the performance of the AM60B alloy with these selected concentrations of graphene reinforcement to better understand its effects.

**Figure 1:**
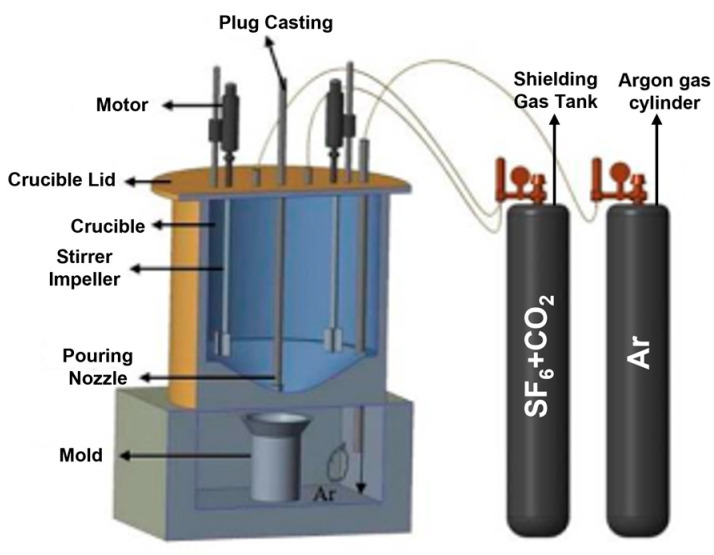
Stir casting schema used in this study [19].

The composites were fabricated using the stir casting method. Initially, AM60B magnesium alloy and graphene particles were placed into a steel crucible, which was then gradually heated to 760 °C. A stabilization period of 15 min was maintained at every 100 °C rise to ensure uniform heating. At 400 °C, a protective gas mixture of carbon dioxide (CO_2_) and sulfur hexafluoride (SF6) was introduced to prevent the magnesium alloy from igniting [16]. As the temperature continued to rise to 700 °C, argon gas was utilized to prevent oxidation. Once the temperature reached 760 °C, the melt was mechanically stirred with two blades at 300 rpm for 5 min, ensuring a uniform dispersion of graphene particles within the AM60B alloy. The molten mixture was then poured into a mold while still in its liquid state and the solidified ingot of the composite was collected from the mold. A schematic of gravity stir casting is presented in Figure 1.

No specific method was implemented to prevent the agglomeration of graphene during fabrication. Instead, the stirring speed of 300 rpm was used to disperse the graphene within the matrix. Although this is not a standardized procedure for stirring speed, many researchers have adopted similar stirring speeds when working with composite materials that include reinforcements [15,17,18]. Graphene exhibits unique properties compared to other reinforcements, and there is limited research on how stirring speed specifically affects its distribution. Thus, the chosen speed of 300 rpm was used to focus on the metallurgical characteristics and mechanical properties produced by this stir casting process.

**Figure 2:**
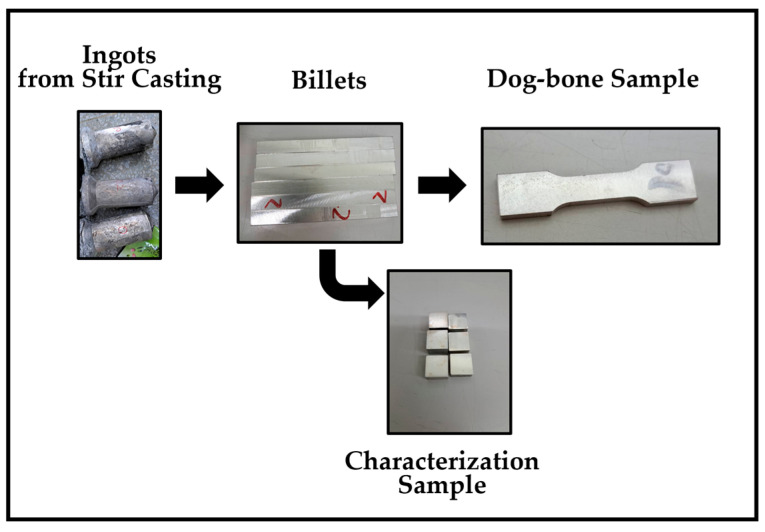
Specimen preparation.

For material characterization, the cast ingots were machined into billets with dimensions of 11 mm × 11 mm × 80 mm. These billets were utilized for various characterization techniques, including X-ray diffraction (XRD), optical microscopy (OM), and scanning electron microscopy (SEM), to analyze phase composition, grain size, and microstructural features. For mechanical testing, the tensile specimens were machined into a dog-bone shape according to ASTM E8-69 [20]. This standard ensures consistent dimensions and geometry of specimens, facilitating accurate and reproducible tensile testing. Dog-bone specimens were prepared to evaluate yield strength, tensile strength, and elongation at break. Figure 2 presents the specimen preparation used in this study.

## 3. Results

### 3.1. Microstructural Characterization

#### 3.1.1. X-ray Diffraction

X-ray diffraction (XRD) was conducted to determine the phase proportions in the multiphase alloys used in this study, as well as to assess the crystallite size and lattice strain resulting from the addition of graphene reinforcement. Since graphene contains carbon, an initial XRD analysis was performed on the reinforcement material to confirm its composition. The XRD patterns were analyzed using a Bruker D2 PHASER X-ray Diffractometer (Bruker Co., Boston, MA, USA) to ensure accuracy in phase identification and crystallographic analysis.

**Figure 3:**
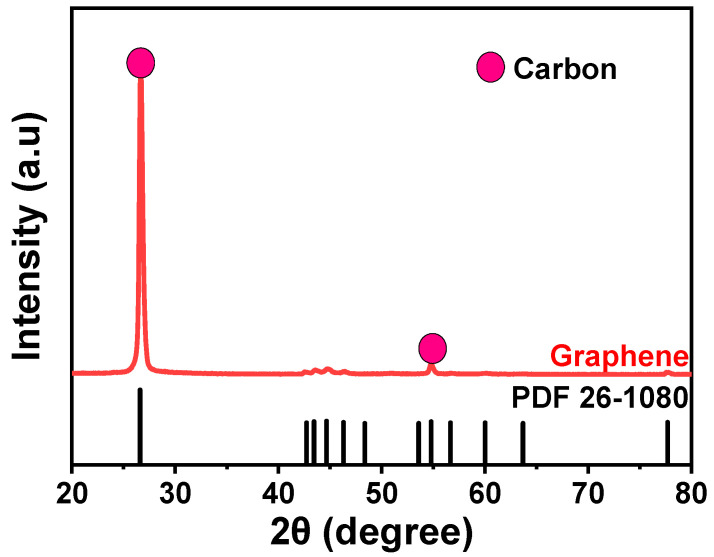
XRD Analysis of Graphene for Reinforcement.

Figure 3 shows the XRD pattern of graphene, which exhibits a distinct and pronounced double peak. These peaks, occurring at 26.6° and 54.79°, correspond to the (004) and (008) planes of the carbon (C) phase, respectively, confirming the presence of graphene [21]. The crystalline structure of graphene was further validated by the close agreement between these peaks with the 2θ value typically seen for the (002) plane at approximately 26.5°.

**Figure 4:**
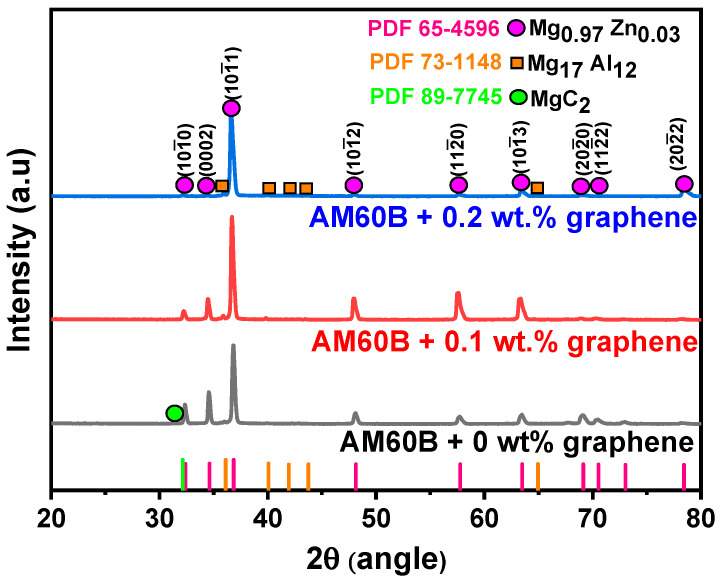
XRD results for all samples.

The XRD analysis of all composite samples confirmed the presence of several phases, as illustrated in Figure 4**,** and was conducted using Diffract EVA V4.3 software. The crystallographic planes for each peak were indexed following the Miller–Bravais system with four indices (hkil). Table 3 displays the peak positions and their corresponding phases. In all samples, the primary phase Mg_0.97_Zn_0.03_ (PDF 65–4596) and the secondary phase Mg_17_Al_12_ (PDF 73–1148) were identified [22]. Additionally, with the incorporation of graphene reinforcement, a new phase, magnesium carbide (MgC_2_), was anticipated based on PDF 89–7745 and supported by previous research [23]. Although the formation of this phase was expected, the graphene phase was difficult to detect in the XRD analysis due to its small concentration. Its absence does not indicate the complete lack of alloying elements; rather, the graphene content falls below the detection sensitivity of the XRD instrument.

However, phase quantification and the index plane are presented in Table 4. The reference intensity ratio method, which is well suited to bulk samples, was employed to determine the number of phases. Note that the sum of the weights of all phases must be equal to 1, and that the quantification values were governed using Equation (1):

(1) $$W_{\alpha }=\frac{Ii\alpha }{RIR\alpha c}\times (\sum_{k=1}^{n}\frac{Iik}{RIRkc})$$

**Figure 5:**
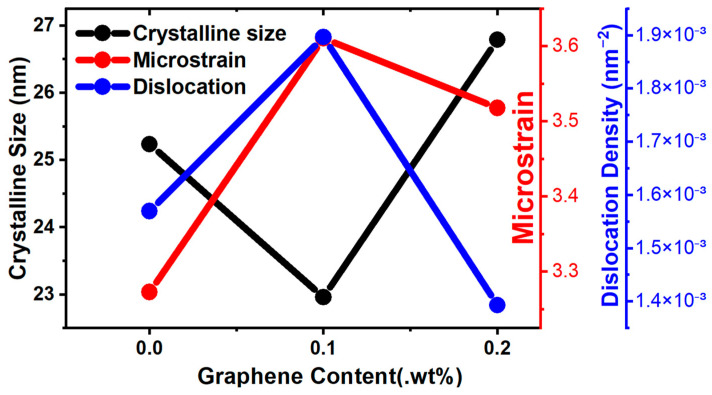
Crystalline size, microstrain, and dislocation density derived from XRD data.

On the other hand, it is important to examine the relationships among crystallite size, crystallinity, microstrain, and dislocation density, all of which can be derived from the XRD patterns. Figure 5 presents the Williamson–Hall plot, which was used to calculate the crystallite size and microstrain. The dislocation density (*δ*) is calculated using Equation (2), where *D* is the crystallite size. The Williamson–Hall plot can be represented using Equation (3):

(2) $$\delta =\frac{1}{D^{2}}$$

*β*Cosθ = *Kλd* + 4εSinθ (3)

where *β* corresponds to the FWHM of each XRD peak, *K* represents the Scherrer constant (typically 0.9), *λ* denotes the wavelength of the X-ray beam (in this study, we used Cu Kα = 0.154060 nm), *d* denotes average crystallite size, ε shows the lattice strain, and θ denotes Bragg’s angle.

Crystallinity is highest when using a graphene percentage of 0.1 wt%. This is mostly related to the intensity shown by the same plane of the peaks from XRD, as shown in Table 5. Equation (4) is used to calculate the crystallinity index, indicating that crystallinity is the ratio of the intensity difference between the crystal peaks and the amorphous peak intensity:

(4) $$Crystallinity\;index\left(CI\right)=\;\frac{Ihkl-Iam}{Iam}\times 100\%$$

#### 3.1.2. Raman Spectroscopy

**Figure 6:**
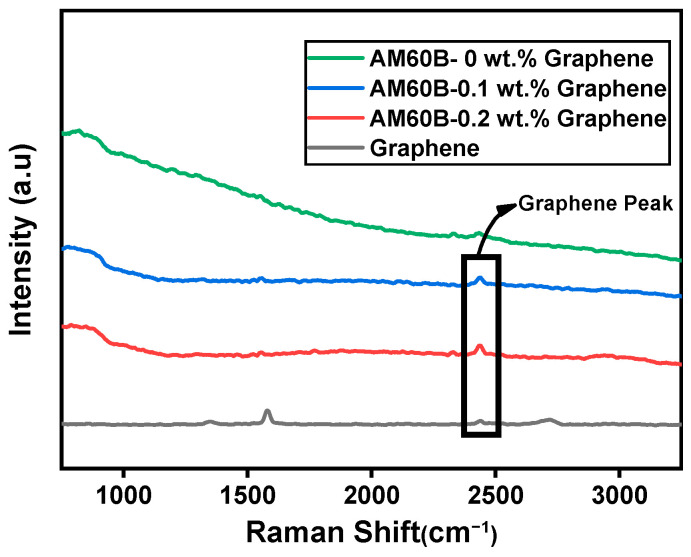
Raman spectroscopy of all samples.

Due to the limitations of sensitivity in XRD analysis, we employed Raman spectroscopy to further investigate the presence of the MgC_2_ and graphene phases. Raman spectroscopy was employed to analyze the carbon content within the material by examining the Raman shifts in the range of 1750–3750 cm^−1^. Raman spectroscopy was conducted using a HORIBA iHR550 spectrometer, equipped with a 532 nm laser. The results (as shown in Figure 6) exhibit a prominent Raman peak around 2435 cm^−1^, which is characteristic of the graphene phase. This observation is consistent with previous studies, which indicate that graphene has distinct Raman-active modes, confirming the presence of the reinforcement material. In contrast, samples without graphene reinforcement did not show significant peaks in this region. This evidence strengthens the hypothesis that the graphene phase is to be present in the reinforced sample. 

#### 3.1.3. Optical Microscopy

**Figure 7:**
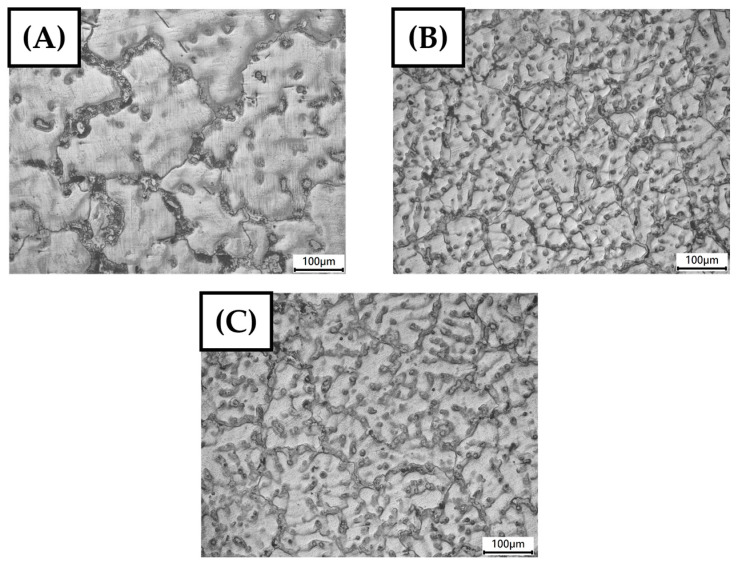
Optical microscopy images of AM60B graphene grains: (**A**) x = 0, (**B**) x = 0.1, and (**C**) x = 0.2.

The microstructural features, particularly grain size, are detailed through optical microscopy (OM) images presented in Figure 7, following the crystalline size analysis from the XRD section. OM analysis was conducted using an Olympus BX41M microscope to capture these images for visual interpretation for the microstructure feature. The graphical representation aligns well with the previous discussion. It is evident from the OM images that grain boundary strengthening occurs through the grain refinement mechanism due to the addition of graphene. This phenomenon is known as the Hall–Petch mechanism, where smaller grain sizes increase the material’s resistance to dislocation motion, enhancing its strength [24,25].

**Figure 8:**
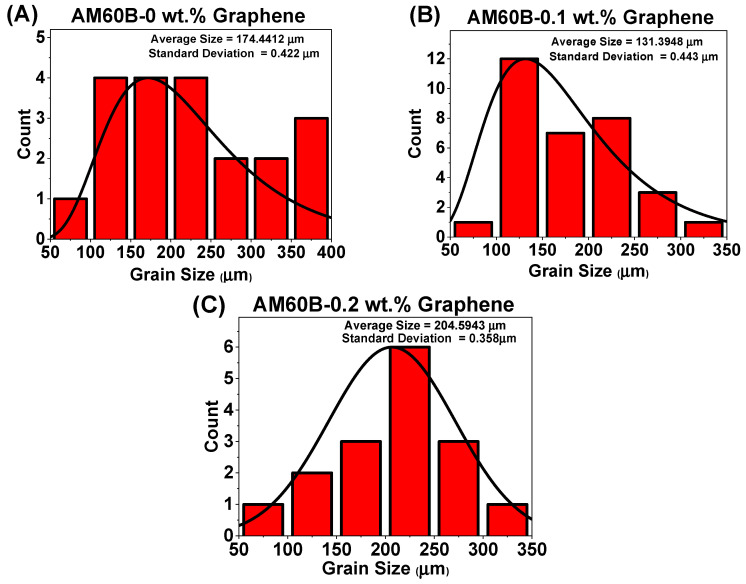
Calculated grain size with the addition of reinforcement AM60B-xGraphene: (**A**) x = 0, (**B**) x = 0.1, and (**C**) x = 0.2.

In this study, the average grain size for all composites was determined using ImageJ^®^ version 1.54k, and the values are shown in Figure 8. The grain size distribution was plotted using a lognormal distribution due to its suitability for representing grain size data, which often exhibit a skewed distribution [26]. This distribution effectively captures the spread of grain sizes and their frequency. The standard deviation is within an acceptable range, confirming that the lognormal distribution is appropriate for calculating the grain size in this context.

It is observed that the average grain size decreases for the AM60B alloy reinforced with 0.1 wt.% graphene, indicating effective grain refinement and strengthening via the Hall–Petch mechanism. However, for the AM60B alloy with 0.2 wt.% graphene, the grain size increases, potentially leading to a reduction in mechanical performance. Previous studies have suggested that there may be a decrease in the Hall–Petch slope at extremely fine grain sizes, depending on the alloying elements and microstructural characteristics [27]. The reduction in grain size, particularly at 0.1 wt.% graphene, suggests that Hall–Petch strengthening plays a key role in enhancing the yield strength of the material.

#### 3.1.4. SEM-EDS Analysis

The fabrication process employed in this study utilized stir casting, where both the matrix and reinforcement materials are melted together. The process temperature was determined based on the matrix material, which was set at 760 °C [28]. Graphene was selected as the reinforcement, composed of carbon—a common element that typically forms interstitial solid solutions due to its smaller atomic size compared to solvent atoms. However, graphene’s unique chemical structure and high melting point, which is higher than that of magnesium alloys, suggest that it may not contribute to solid solution strengthening. Instead, graphene likely enhances the material through other strengthening mechanisms, which will be discussed later in the study. To further investigate the material’s microstructure, SEM-EDS analysis was conducted using a JEOL 7900F FE-SEM (Tokyo, Japan).

**Figure 9:**
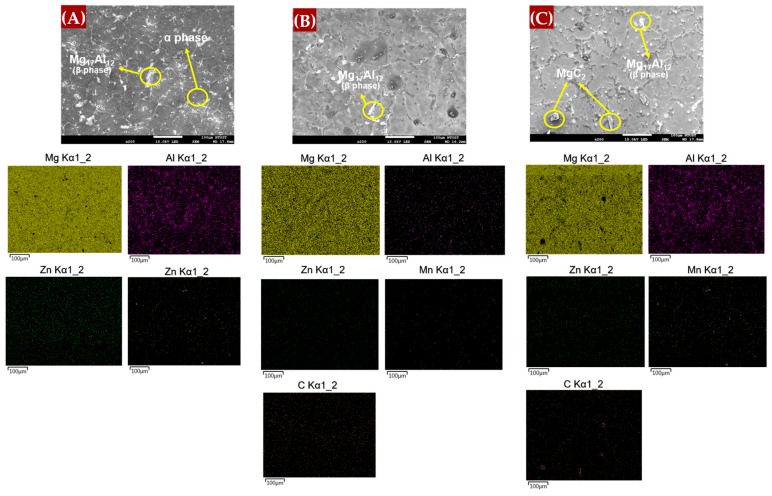
SEM-EDS of AM60B/graphene composites with (**A**) 0 wt.%, (**B**) 0.1 wt.%, and (**C**) 0.2 wt.%.

**Figure 10:**
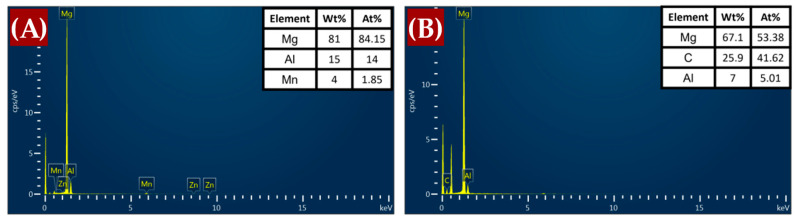
EDS points of the phase formed in the sample (**A**) Mg_17_Al_12_; (**B**) MgC_2_.

Figure 9 presents the energy-dispersive X-ray spectroscopy (EDS) mapping images for the AM60B/graphene composites with (A) 0 wt.%, (B) 0.1 wt.%, and (C) 0.2 wt.%. These images offer insights into the elemental distribution and the effects of non-metallic reinforcement inclusions. The scanning electron microscopy (SEM) observations revealed the presence of secondary Mg_17_Al_12_ intermetallic phases, which are formed with the addition of alloying elements. For the 0.1 wt.% graphene sample, the MgC_2_ phase is less likely to form and remains undetectable in XRD analysis (as shown in Figure 4), likely because it is below the detection limit [14]. However, at 0.2 wt.% graphene, there is evidence of carbon agglomeration in specific surface areas, which may correspond to MgC_2_ formation. The composition of the agglomerates is further clarified by the EDS point analysis in Figure 10.

### 3.2. Mechanical Testing

#### 3.2.1. Tensile Testing

**Figure 11:**
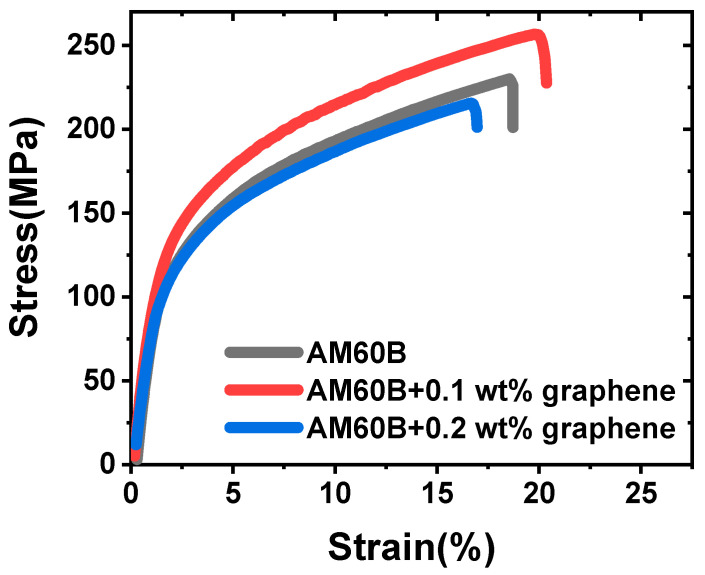
Tensile stress–strain curve of all samples.

To evaluate the mechanical properties of the fabricated composites, tensile tests were performed using a universal testing machine (MTS Insight Electromechanical 10 kN, MTS Systems Corporation, Eden Prairie, MN, USA) at room temperature (25 °C) with a strain rate of 0.5 mm/min. For each material composition, three samples were tested, and the resulting tensile curves were averaged and presented in Figure 11. Key mechanical properties, including yield strength, ultimate tensile strength, and strain at break, were determined and are summarized in Table 6, along with a comparison to previous studies.

It is important to note that the data in Table 6 are not limited to the specific type of graphene powder used in this study, as there are various forms of graphene that can be used as reinforcements, such as graphene nanosheets, graphene nanoplatelets, and graphene oxide [29,30]. Although the types of graphene differ, these comparisons provide useful references for understanding their effects on mechanical properties.

As observed in prior studies, the use of graphene oxide and graphene nanoplatelets typically enhances the mechanical properties of composites as the graphene content increases. However, with graphene nanosheets, the mechanical properties can vary, sometimes decreasing when higher graphene percentages are used. This phenomenon highlights the need for further investigation, including metallography and characterization, to understand the underlying mechanisms. Similarly, in this study, the optimal graphene content for improving mechanical properties was found to exist within a specific range. Beyond this range, increasing the graphene content does not necessarily yield better results and can even have adverse effects.

Notably, the yield strength of the composites was highest when 0.1 wt.% graphene was employed, showing an increase of approximately 13.4% compared to the sample without reinforcement. However, upon the addition of 0.2 wt.% graphene, the yield strength began to decrease by approximately 8.3%, along with reductions in other mechanical properties such as ultimate tensile strength and strain at break. This decrease may be attributed to the higher reinforcement content, which tends to make the metal less ductile compared to that with lower graphene content [31].

**Table 6:** Comparison of some previous studies with the result in this study on the mechanical properties of magnesium alloys fabricated using the casting process.

Material	Yield Strength (MPa)	Ultimate Tensile Strength (MPa)	References
AZ31	183 ± 4.3	267 ± 6.5	[32]
AZ31-1.5GNP	187 ± 3.5	284 ± 5.4	
AZ31-3GNP	195 ± 4.5	299 ± 6.2	
ZK61	187 ± 1	307 ± 1	[33]
ZK61-0.1RGO	191 ± 2	307 ± 0	
ZK61-0.25RGO	195 ± 1	298 ± 1	
ZK61-0.4RGO	200 ± 2	301 ± 0	
ZK61-0.6RGO	203 ± 2	312 ± 3	
AZ61 alloy	184 ± 5.5	300 ± 7.1	[34]
AZ61-3GNP	232 ± 5.5	335 ± 9.1	
AZ80	104 ± 5.2	271 ± 13.5	[35]
AZ80-0.1GNP	146 ± 7.3	310 ± 15.5	
AZ80-0.2GNP	160 ± 16	325 ± 32	
AZ91(T4)	168 ± 5.0	215 ± 6.0	[36]
AZ91-0.1GNS	223 ± 3.6	276 ± 4.2	
AZ91-0.3GNS	268 ± 4.6	318 ± 5.0	
AZ91-0.5GNS	296 ± 3.7	335 ± 4.8	
AZ91-0.8GNS	252 ± 5.5	307 ± 5.0	
AZ91-1.2GNS	234 ± 3.0	287 ± 5.0	
AM60B	101.7 ± 4.9	229.4 ± 4.0	This Study
AM60B-0.1 wt.% graphene	115.3 ± 5.6	256.7 ± 3.7	
AM60B-0.2 wt.% graphene	93.2 ± 4.1	215.4 ± 5.5	

#### 3.2.2. Microhardness Testing

**Figure 12:**
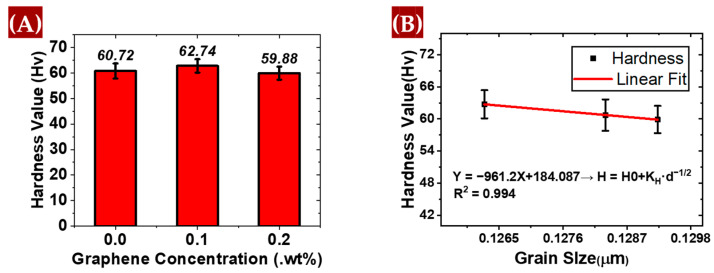
(**A**) Microhardnesses of the alloys and composites, (**B**) Plot hardness vs. grain size for all the samples.

Figure 12A shows that the microhardness of the material slightly increased with the addition of 0.1 wt.% graphene, indicating a marginal improvement in hardness. However, upon further addition of 0.2 wt.% graphene, a noticeable decrease in microhardness is observed. This decline suggests that while a small amount of graphene can enhance hardness, excessive amounts may reduce material strength. The microhardness of the AM60B alloys was measured using a Wilson VH1102/1202 Knoop/Vickers hardness tester. The measurements were conducted with a 300 gf load applied for 10 s using VHPro Express DiaMet VH1102 software to assess the hardness of the material accurately.

The grain size of the sample containing 0.2 wt.% graphene is larger compared to the other samples, which typically leads to lower hardness due to the reduced effectiveness of grain boundary strengthening. Grain boundary strengthening becomes more effective as the grain size decreases because a higher number of grain boundaries hinders dislocation motion, contributing to greater hardness. The observed drop in microhardness may also be attributed to the inhomogeneous distribution of graphene, which could result in areas within the grains that are inadequately reinforced [37]. 

As shown in Figure 12B, the plot of hardness values against grain size shows a linear relationship. As the grain size decreases, hardness increases, which is consistent with the Hall–Petch mechanism, where grain boundary strengthening contributes to improved mechanical properties [38]. This relationship arises because the grain boundaries tend to exhibit higher hardness compared to the interior of the grains, which helps in terms of impeding dislocation movement and improving overall hardness.

### 3.3. Regression Analysis Based on Mechanical Testing

**Figure 13:**
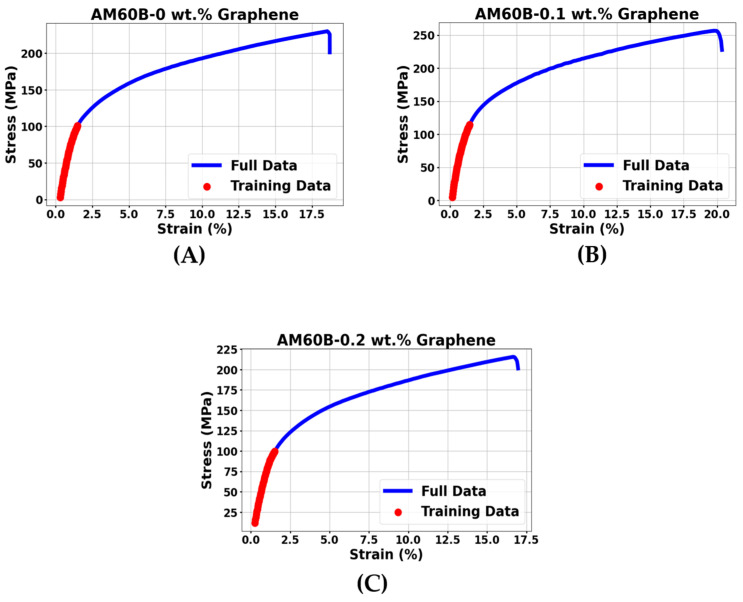
Training data for regression analysis AM60B-xGraphene: (**A**) x = 0, (**B**) x = 0.1, and (**C**) x = 0.2.

In this study, a machine learning approach was employed to analyze the elastic regions of the stress–strain curves obtained from the tensile tests of AM60B magnesium alloy composites, as illustrated in Figure 13. Previous studies have explored both experimental mechanics and the application of machine learning to analyze stress–strain curves [38] and optimize material composition [39]. The objective of this study was to use machine learning regression algorithms, specifically linear regression, to model the relationship between stress and strain within the elastic region.

The elastic region is crucial because it represents the initial linear portion of the stress–strain curve. By accurately modeling this region, regression analysis can offer valuable insights into the elastic properties of the composite, such as the modulus of elasticity, and predict the material’s behavior under small deformations. The regression model was trained using the full dataset derived from the stress–strain curves of the AM60B magnesium alloy composites. To ensure the model’s validity, this study implemented an 80–20 split of the data, with 80% allocated for training and 20% for validation. This approach allowed us to test the model on unseen data and verify that it did not overfit the training set [40,41].

Although hyperparameter tuning is an important step in optimizing machine learning models, including linear regression, its impact can vary depending on the algorithm and dataset [42]. In some cases, the default parameters may yield satisfactory results, and further tuning may provide only marginal gains. As the current regression analysis performed well within the elastic region, extensive hyperparameter optimization was not pursued.

**Figure 14:**
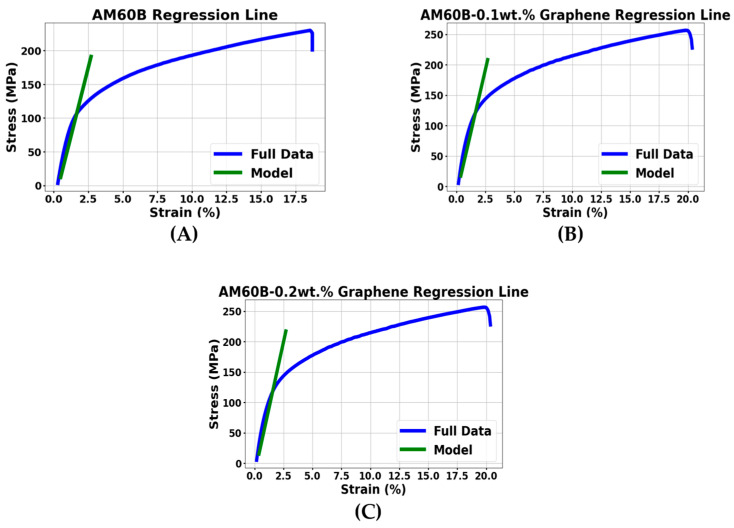
Regression lines of AM60B-xGraphene: (**A**) x = 0, (**B**) x = 0.1, and (**C**) x = 0.2.

The machine learning regression analysis was performed by fitting a linear model to the data points within the elastic region. This model allows for the prediction of stress based on strain values, which is fundamental to understanding the mechanical behavior of materials. To evaluate the performance of the regression model, various machine learning metrics, such as R-squared (R^2^) and mean absolute error (MAE), were calculated. These metrics help determine the accuracy and reliability of the model, ensuring that the predictions closely match the experimental data. As shown in Figure 14, which presents the regression lines generated for the elastic region, the green regression lines fit well within the elastic region for all three compositions. The accuracy and performance of the regression model will be assessed using specific machine learning metrics, which will be discussed in detail in the later sections of this study.

## 4. Discussion

### 4.1. Strengthening Mechanism

When studying the impact of small reinforcement additions on mechanical properties, it is crucial to consider the various strengthening mechanisms at play. These mechanisms, which influence the dislocation motion, directly affect material strength. As the dislocation mobility decreases, the material strength increases [43]. Strengthening mechanisms can be categorized into several types, including obstacle strengthening (Orowan), the Hall–Petch effect, thermal expansion mismatch (CTE), and load transfer strengthening. Each mechanism plays a distinct role in enhancing the mechanical properties of the material [44].

Obstacle strengthening, also known as Orowan strengthening, occurs when dislocations encounter obstacles such as precipitates or particles that impede their motion. The interaction between the strain field of a dislocation and these obstacles increases the material strength by increasing the energy required for dislocation movement. The length of the dislocation is directly related to the strain energy introduced into the material, which can be expressed as the elastic energy per unit length. This energy ($E_{⊥})$ is expressed by Equation (5) involving the shear modulus (*μ*), the magnitude of the Burgers vector (*b*), the radius of influence around the dislocation (*R*), and the core radius of the dislocation (*r_o_*) [45,46]:

(5) $$E_{⊥}=\frac{\mu b^{2}}{4\pi }ln\frac{R}{r_{o}}$$

From the concept of elastic energy, the relationship can be further extended to calculate yield strength. Using the Orowan–Ashby equation, it is important to note that some of the symbols in the equation may differ. In this context, *b* represents the Burgers vector, *G_m_* is the shear modulus, *d_p_* denotes the mean particle size, and *V_f_* refers to the volume fraction, which can be calculated using Equation (6). The symbol *λ* represents the interparticle distance between graphene particles, which can be determined using Equation (7). Once *λ* is found, Equation (8) can then be applied to calculate the Orowan strengthening contribution:

(6) $$V_{f}=\frac{W_{R}\times \rho_{R}}{W_{m}\times \rho_{m}}$$

(7) $$\lambda =d_{p}(\frac{1}{V_{f}^{1/3}-1})$$

(8) $$∆\sigma_{o}=\frac{0.13G_{m}b}{\gamma }\ln\left(\frac{d_{p}}{2b}\right)$$

**Figure 15:**
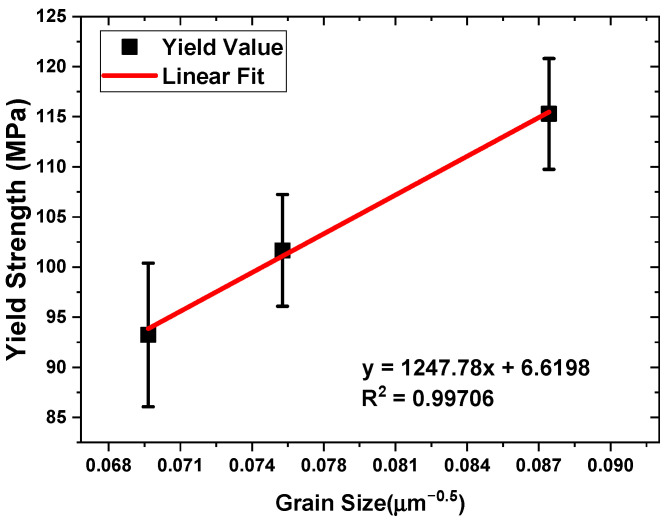
Linear fit yield strength vs. grain size.

Another strengthening mechanism is grain boundary strengthening, known as the Hall–Petch effect, shown in Equation (9), which describes the relationship between the yield strength of the composite (*σ_y_*), material-specific constants (*σ_o_* and *K*), and grain size (*d*) [47]. The constant *K* can be determined by plotting the yield strength as a function of the inverse square root of the grain size (*d*) in Figure 15.

Despite having only three samples, the plot shows that the R^2^ value is within an acceptable range, indicating a reliable fit. This strengthening mechanism is distinct from obstacle strengthening because it specifically focuses on the influence of grain size and boundaries on material strength [48]. The Hall–Petch equation can be further expanded and expressed in Equation (9) to determine the extent to which the grain size contribution affects the yield strength:

(9) $$\sigma_{y}=\sigma_{0}+Kd^{-\frac{1}{2}}$$

(10) $$\Delta \sigma_{\mathrm{HP}}=K\left(d_{\mathrm{composite}}^{-\frac{1}{2}}-d_{\mathrm{AM}60B}^{-\frac{1}{2}}\right)$$

(11) $$\Delta \sigma_{\mathrm{CTE}}=\alpha G_{m}b\left(\sqrt{\frac{BV_{f}\Delta \mathrm{CTE}\Delta T}{bd_{p}(1-V_{f})}}\right)$$

The final strengthening mechanism considered in this study is load transfer (LT). LT tends to contribute significantly to the overall strengthening of composite materials, primarily because it is directly related to the yield strength of the material [49]. The load transfer equation is given by Equation (12). In composite systems, the reinforcement, such as graphene, bears part of the applied load, thereby reducing the stress on the matrix and effectively increasing the composite’s overall yield strength. This mechanism becomes particularly important when the reinforcement exhibits a much higher stiffness and strength than the matrix, allowing it to carry a substantial portion of the load and thereby enhancing the mechanical properties of the entire composite [50]:

(12) $$\Delta \sigma_{LT}=\frac{S\sigma_{m}V_{f}}{4}$$

All the constants used for the strengthening mechanisms are compiled in Table 7. Some constants are derived from the experimental data in this study, while others are taken from references. Due to the complexity of the equations, Table 8 provides a summary of the calculation procedures. After performing the calculations, Table 9 presents the contributions of each strengthening mechanism.

The purpose of these tables is not to directly compare the mechanisms for each composition, but rather to highlight which mechanisms contribute significantly more and which contribute less. Among the two compositions studied, the load transfer mechanism is the most significant contributor to yield strength, as expected, since it is directly proportional to the material’s yield strength.

The second-most influential mechanism is the Hall–Petch effect, related to the grain boundaries observed in all samples. While this mechanism strengthens the material, its impact is less than that of load transfer. Orowan strengthening is the third-most significant mechanism, likely less pronounced due to the lower volume fraction of reinforcement in the compositions. Finally, the coefficient of thermal expansion (CTE) mismatch contributes the least, which is reasonable given that no heat treatment was applied to enhance its effects.

### 4.2. Fracture Surface Study

**Figure 16:**
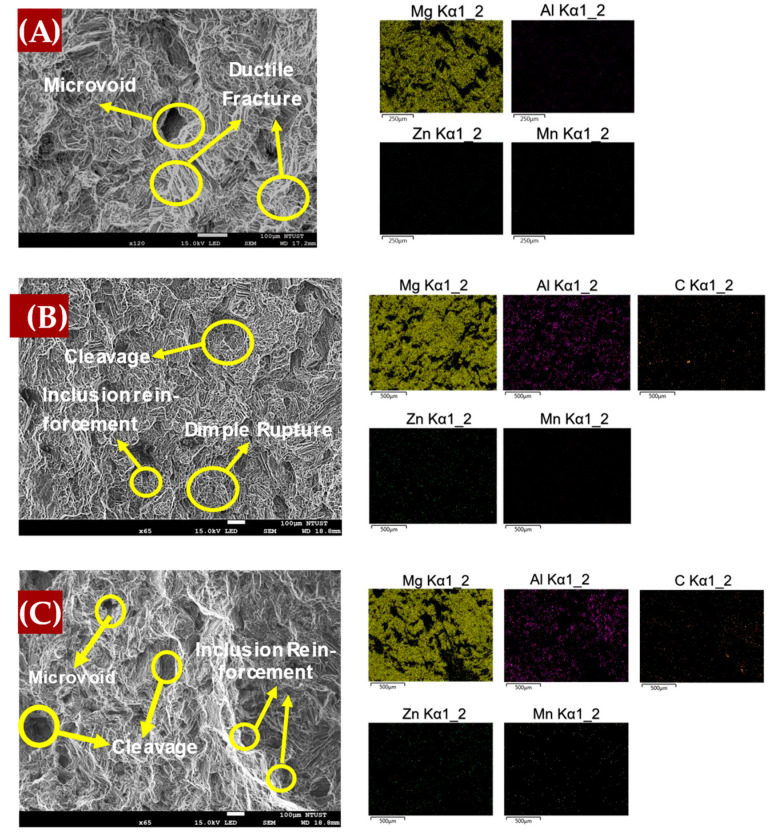
Fracture Surface Study of AM60B-xGraphene (**A**) x = 0, (**B**) x = 0.1, (**C**) x = 0.2.

The fracture surfaces of the samples used in this study were analyzed to understand the failure mechanisms during tensile testing. Typically, fracture surfaces can be classified into two main categories: brittle and ductile. Each of these fracture types revealed important information about the material’s behavior under stress, particularly in relation to the microstructural features of the composite. For the as-cast 0 wt.% graphene sample, the SEM images in Figure 16A reveal distinct microvoids and dimples characteristic of ductile fracture behavior. These microvoids suggest that the material underwent significant plastic deformation before failure. The presence of cup-like structures further corroborates the ductile nature of this sample, indicating that the material retains a more ductile response under tensile stress.

In contrast, the introduction of 0.1 wt.% graphene, as observed in Figure 16B, resulted in a noticeable shift toward brittle fracture behavior. The SEM images exhibit clear cleavage planes marked by river patterns, which indicate a brittle fracture mechanism. This suggests that even a small addition of graphene can induce brittleness in the material. Moreover, inclusions of non-metallic reinforcement, identified as graphene with elemental carbon content, were evident in the EDS images. As the graphene content increased to 0.2 wt.%, as shown in Figure 16C, the fracture surface analysis revealed a mix of ductile and brittle features. While microvoids were still present, indicating some ductile behavior, the increased prevalence of cleavage planes suggested a stronger tendency toward brittleness. This behavior can be attributed to the higher graphene content, which may lead to the agglomeration of graphene particles, which act as stress concentrators and reduce the overall ductility of the material.

**Figure 17:**
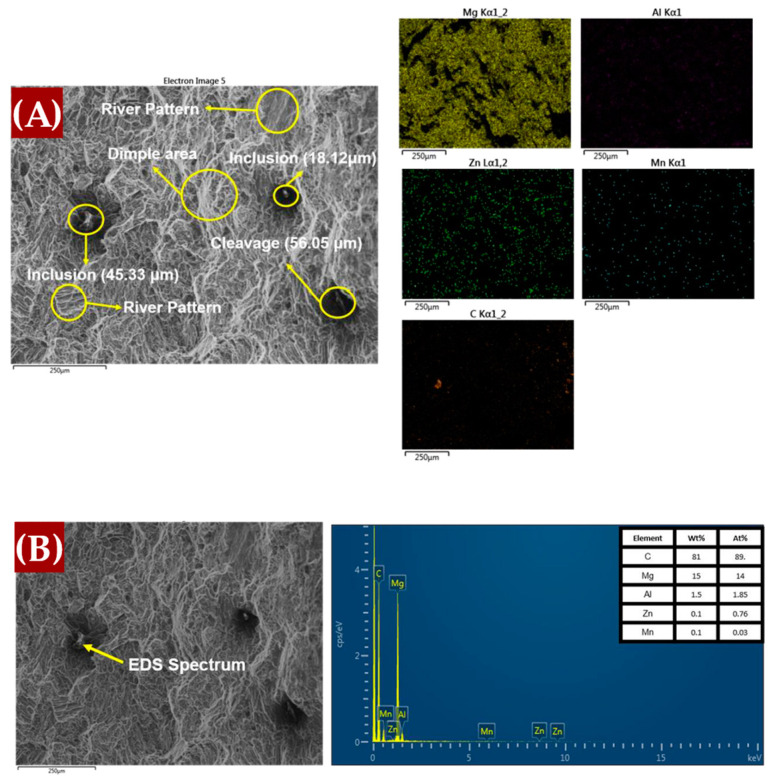
Additional SEM-fracture surface of AM60B-0.2wt.%. (**A**) EDS mapping. (**B**) EDS spectra.

As observed, the 0.2 wt.% graphene addition tends to increase the brittleness of the material compared to other concentrations. Consequently, further SEM-EDS analysis was conducted to better understand the fracture behavior at this level of graphene content. Figure 17 provides a detailed examination of the fracture surface for the 0.2 wt.% graphene composite. In Figure 17A, EDS mapping highlights the presence of graphene agglomerations, as evidenced by the elevated carbon content. The EDS element maps confirm the presence of carbon-rich regions, indicative of graphene clusters, which likely contribute to the observed brittleness by acting as stress concentrators [57]. Figure 17B shows the EDS spectrum, which reveals a high percentage of carbon, confirming the presence of graphene within the material. This finding supports the notion that the increased graphene content leads to agglomeration, thus reducing ductility and promoting brittle failure mechanisms.

### 4.3. Regression Analysis Using Machine Learning Metrics

**Figure 18:**
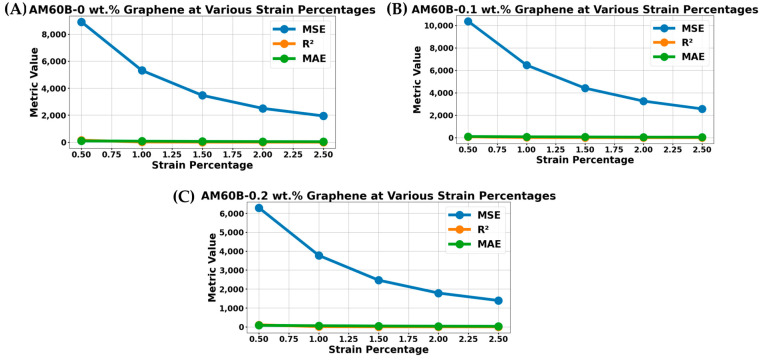
MSE, R^2^, and MAE for the stress–strain curve AM60B-xGraphene: (**A**) x = 0, (**B**) x = 0.1, (**C**) x = 0.2.

Figure 18 illustrates the evaluation metrics used in the regression analysis of the stress–strain curves for the samples in this study. From Figure 18A–C, it is evident that all machine learning metrics decrease as the strain percentage increases. This trend indicates that regression analysis becomes increasingly accurate as the strain percentage increases. The exact values of these metrics are given in Table 6, Table 7 and Table 8.

Interestingly, the mean squared error (MSE) was higher for the sample with 0.1 wt.% graphene reinforcement than for the sample without reinforcement. However, the sample with 0.2 wt.% graphene exhibited the highest MSE, suggesting that the machine learning model found it more challenging to converge with increasing reinforcement. This could be due to the complexity introduced by the reinforcement, which may have affected the model’s ability to accurately predict the stress–strain relationship. Moreover, the R^2^ metric, which is negative in some cases, indicates that the model does not adequately capture variance in the data.

Despite this, Table 10 shows that the evaluated strain was 0.5 %, which is in the elastic region. The evaluated elastic modulus varied, with AM60B with 0.1 wt.% graphene exhibiting the highest. Note that the machine learning metric for all samples is the AM60B model without reinforcement, featuring minimum error. The mean absolute error (MAE) follows a similar trend to the MSE, further highlighting the challenges faced by the regression model with different reinforcement levels. This study shows that regression analysis, particularly linear regression, can be a useful tool for understanding the material behavior in the elastic region. Materials may not always exhibit perfectly linear behavior in this region, and slight deviations can occur.

## 5. Conclusions

This study evaluates the impact of incorporating graphene as a reinforcement in AM60B magnesium alloy, revealing important insights into the relationship between graphene content and alloy mechanical properties.

- Incorporating 0.1 wt.% graphene into the AM60B magnesium alloy resulted in a significant improvement in yield strength, achieving a 12% increase compared to the alloy without graphene. These results indicate that a modest addition of graphene effectively enhances the alloy’s mechanical performance.
- The addition of 0.1 wt.% graphene not only enhanced the yield strength by approximately 13.4% but also increased the strain at break by 8.8%. This suggests that a small amount of graphene slightly improves both the strength and ductility of the alloy, making it more robust and versatile for various applications.
- However, increasing the graphene content to 0.2 wt.% reduced the tensile strength. This decline was likely due to the agglomeration of graphene particles and the resulting increase in brittleness, which compromised the alloy’s overall mechanical properties.
- Regression analysis of the tensile curves showed that the addition of 0.1 and 0.2 wt.% graphene slightly reduced the model accuracy, as reflected by the mean squared error (MSE) of approximately 0.065. However, the accuracy remained high, indicating the model’s robustness despite the challenges posed by the reinforcement.
- The thermal stability of AM60B/graphene composites, particularly in relation to the formation of the MgC_2_ phase, was not assessed in this study. Given that graphene is well-regarded for its excellent thermal stability, investigating this aspect could provide valuable insights into how it influences the thermal behavior of the composites.
- In future work, the exploration of more complex regions of the stress–strain curve, such as the plastic region, may benefit from hyperparameter tuning to improve model performance.

## Figures and Tables

**Table 1:** Elemental composition of the matrix material in wt.%.

Elements	Al	Mn	Zn	Si	Fe	Cu	Ni	Mg
wt.%	5.800	0.320	0.220	0.100	0.005	0.01	0.002	Bal

**Table 2:** Compositions of the composites used in this study.

No.	Matrix (AM60B) (wt.%)	Reinforcement (Graphene) (wt.%)
1	100	0
2	99.9	0.1
3	99.8	0.2

**Table 3:** Peak positions and the associated phases.

Associated Phases	Peak Positions
Mg_0.97_Zn_0.03_	32.391
	34.611
	36.854
	48.131
	57.776
	63.499
	67.812
	69.13
	70.525
	73.017
	78.424
Al_12_Mg_17_	36.12
	40.079
	41.943
	43.742
	64.94
MgC_2_	32.15

**Table 4:** Phase quantification in this study.

Sample	Associated Phase	Pattern	Percentage (%)	Structure
AM60B 0 wt.% graphene	Mg_0.97_Zn_0.03_	PDF 65–4596	81.4	Hexagonal
	Mg_17_Al_12_	PDF 73–1148	18.6	Cubic
AM60B 0.1 wt.% graphene	Mg_0.97_Zn_0.03_	PDF 65–4596	87.7	Hexagonal
	Mg_17_Al_12_	PDF 73–1148	8.9	Cubic
	MgC_2_	PDF 89–7745	3.4	Tetragonal
AM60B 0.2 wt.% graphene	Mg_0.97_Zn_0.03_	PDF 65–4596	83.6	Hexagonal
	Mg_17_Al_12_	PDF 73–1148	11.3	Cubic
	MgC_2_	PDF 89–7745	5.1	Tetragonal

**Table 5:** Estimated crystalline size, dislocation density, and microstrain from XRD analysis.

Samples	Crystalline Size (nm)	Microstrain	Dislocation Density (nm^−2^)
AM60B	25.237	3.273	0.00157
AM60B-0.1 Gr	22.96	3.611	0.0019
AM60B-0.2 Gr	26.79	3.518	0.00139

**Table 7:** Parameters for calculating the strengthening mechanism.

Parameter	Definition	Value	References
*K*	Hall–Petch Constant	1247.78 MPa μm^0.5^	Calculated
*V_f_*	Volume Fraction of Reinforcement	0.077% and 0.154%	Calculated and [51]
*S*	Aspect Ratio	1272	[52]
*V_m_*	Volume Fraction of Matrix	99.9232% and 99.9846%	Calculated
*σ_m_*	Yield Strength of Matrix	101.665 MPa	Experimental
*α*	Thermal Constant	1.25	[52]
*G_m_*	Shear Modulus	8.132 GPa	Experimental
*b*	Burgers Vector	0.32 nm	[53]
*B*	Geometric Constant	12	[54]
Am	Thermal Expansion of Matrix	25:2 × 10^−6^ C^−1^	[55]
Ar	Thermal Expansion of Reinforcement	−8 × 10^−6^/°C	[56]
Tt	Testing Temperature for Tensile	25 °C	Temperature during Testing
*d_p_*	Reinforcement Size	3.9 µm	Calculated from Purchased Certificate

**Table 8:** Summary of strengthening mechanism and association equations.

Strengthening Mechanism	Associating Equation
Orowan Strengthening	Equation (6); the rest of the constants are tabulated in Table 4
Hall–Petch	Equation (7) to plot the linear fit and then Equation (8) to calculate the Hall–Petch value
Load Transfer	Use the value of yield strength matrix (*σ_m_*), the aspect ratio (S) from reference and calculate (*V_f_*) from the equation (use Equation (10) with respect to the constant in Table 7)
CTE	Directly use Equation (9) with respect to the constant in Table 7

**Table 9:** Each strengthening mechanism contribution.

Materials	HP	LT	CTE	Orowan
AM60B 0.1 wt%	14.3869	28.23015426	0.001699	0.078315446
AM60B 0.2 wt%	7.237	45.65757504	0.002376	0.028294488

**Table 10:** Evaluation of the modulus elasticity compared with the machine learning metric for each sample.

Material	Evaluated Strain (%)	MSE	R^2^	MAE	E(MPa)
AM60B	0.5	0.010509	0.999486	0.098703	124.000253
AM60B-0.1 wt.% Graphene	0.5	0.065118	0.999184	0.21435	135.742772
AM60B-0.2 wt.% Graphene	0.5	0.004508	0.999808	0.058588	104.550762

## Data Availability

The raw data supporting the conclusions of this article will be made available by the authors upon request.
